# Virtual reality exposure therapy for reducing social anxiety in stuttering: A randomized controlled pilot trial

**DOI:** 10.3389/fdgth.2023.1061323

**Published:** 2023-02-09

**Authors:** Ian Chard, Nejra Van Zalk, Lorenzo Picinali

**Affiliations:** ^1^ Design Psychology Lab, Dyson School of Design Engineering, Imperial College London, London, United Kingdom; ^2^ Audio Experience Design Group, Dyson School of Design Engineering, Imperial College London, London, United Kingdom

**Keywords:** social anxiety, stuttering, stammering, virtual reality, exposure, VRET

## Abstract

We report on findings from the first randomized controlled pilot trial of virtual reality exposure therapy (VRET) developed specifically for reducing social anxiety associated with stuttering. People who stutter with heightened social anxiety were recruited from online adverts and randomly allocated to receive VRET (*n* = 13) or be put on a waitlist (*n* = 12). Treatment was delivered remotely using a smartphone-based VR headset. It consisted of three weekly sessions, each comprising both performative and interactive exposure exercises, and was guided by a virtual therapist. Multilevel model analyses failed to demonstrate the effectiveness of VRET at reducing social anxiety between pre- and post-treatment. We found similar results for fear of negative evaluation, negative thoughts associated with stuttering, and stuttering characteristics. However, VRET was associated with reduced social anxiety between post-treatment and one-month follow-up. These pilot findings suggest that our current VRET protocol may not be effective at reducing social anxiety amongst people who stutter, though might be capable of supporting longer-term change. Future VRET protocols targeting stuttering-related social anxiety should be explored with larger samples. The results from this pilot trial provide a solid basis for further design improvements and for future research to explore appropriate techniques for widening access to social anxiety treatments in stuttering.

## 1. Introduction

Stuttering is a developmental speech disorder which disrupts the fluent production of speech (1). Compared to fluent speakers, people who stutter (PWS) are at a greater risk of developing heightened levels of social anxiety (2, 3). Social anxiety is defined as “a marked, or intense, fear or anxiety of social situations in which the individual may be scrutinized by others” (1). Underlying this experience is fear of negative evaluation, and the overestimation of the consequences this will have (4). For approximately 46% of PWS, social anxiety is so severe that it constitutes a clinical diagnosis of social anxiety disorder (5). For these individuals, anxiety is often perceived as persistent and overwhelming. Similar patterns are found amongst children and adolescents who stutter (6), with approximately one third meeting diagnostic criteria for social anxiety disorder (7).

While social deficits are mostly theorized to be consequences of anxiety (4, 8), stuttering itself can manifest as a social performance deficit and be the source of continued negative evaluation from others. As such, anxiety is embedded in speech and revolves around fears of others’ negative reactions to stuttering (9). Speaking on the telephone is one of the most commonly reported fears, to the extent that it may constitute its own sub-type of social anxiety among PWS (10, 11). Avoidance of certain words and situations is also a core theme in the lived experience of stuttering (12). The cognitive-behavioral processes that underlie the maintenance of social anxiety are also modified for the experience of stuttering (9). The experience of comorbid stuttering and social anxiety often results in greater speech dissatisfaction, psychological problems and general negative impact on life (13).

Speech and language therapy is the first port of call for treatment-seeking PWS, but this has limited effects on reducing social anxiety (14). Considering the high rates of social anxiety disorder among PWS and the unique nature of social anxiety experienced by this group, interest in developing stuttering-specific treatments for social anxiety has grown. Most available treatments are based on Cognitive Behavioral Therapy (CBT), which targets the maladaptive thoughts and beliefs that underlie the persistence of social anxiety. CBT is considered the gold-standard in anxiety treatment (15) and has broad support for its use in social anxiety disorder (16). To date, three randomized trials of CBT tailored for PWS have been conducted. The earliest of these found CBT was associated with decreased social anxiety and elimination of social anxiety disorder diagnoses (14). A later study compared the same in-person protocol to an automated, online version of CBT (17). Findings showed both treatments were equally as effective at reducing symptoms of social anxiety. The third study adopted the same automated CBT protocol, though found that it did not improve social anxiety symptoms (18). However, it did find that supplementing automated CBT into speech therapy improved speech outcomes (18), unlike *in vivo* CBT (14). By considering the stuttering-specific nature of social anxiety, these CBT protocols ensure maximum relevance to the lived experience of PWS.

Exposure therapy is an integral part of CBT that might be as efficacious as a standalone treatment (19). Exposure involves patients encountering anxiety-inducing situations alongside corrective information to activate and modify fear structures within memory. The *Emotional Processing Theory* (20) approach employs repeated exposure to feared stimuli to achieve habituation. The resulting lower anxiety response is assumed to represent information that is incongruent to existing fearful associations, thus overwriting fear within memory. In contrast, the *Inhibitory Learning Model* (21) suggests corrective information should consist of exposure stimuli that are inconsistent with individual beliefs and expectations. New associations with feared stimuli are then learnt, thus inhibiting previous fearful associations rather than overwriting them. Inhibitory learning techniques are likely to be highly appropriate for social anxiety treatment in stuttering, as the approach outlines techniques to reduce the risk of fear reacquisition (e.g., occasional fear reinforcement) (22). PWS are at a greater risk of fear reacquisition due to continued reactions to speech. Common stuttering-specific safety behaviors such as avoidance of certain words and internal rehearsal of speech (23, 24) could also be targeted within exposure scenarios, to allow for greater learning of non-threat associations (22).

Typically, exposure exercises targeting social anxiety are created by employing interaction with real people. However, the prospect of conducting exposure virtually has recently become more viable (25–27). Virtual Reality Exposure Therapy (VRET) uses a head-mounted display that virtually recreates environments and characters that replicate anxiety-inducing social situations. Cost-saving through creating standardized exposure scenarios that do not require actors has the potential to reduce public health disparities and widen access to treatment. Additionally, eliminating in-person interaction within exercises may improve the approachability of treatment (28) and treatment-seeking behavior, which is typically low among individuals with social anxiety disorder (29). Using virtual reality (VR) to conduct exposure may also aid efforts to further integrate psychological support into speech therapy by removing much of the burden of conducting *in vivo* exposure exercises. Greater alignment between these two treatments would help to remove barriers to receiving psychological support (30) to increase treatment uptake. It may also help solidify the maintenance of benefits from speech therapy, which can suffer from experiencing comorbid stuttering and mental health conditions (18, 31). Lastly, it would offer PWS the cognitive and behavioral support required to manage stuttering (32).

Using consumer VR and automating VRET by replicating therapist functions virtually could further increase the applicability for PWS. Self-guided treatments are likely to play a greater role in mental health provision, particularly following the Covid-19 pandemic, which saw 54% of countries worldwide adopt digital and self-guided interventions in place of in-person services (33). Benefits include reduction of travel barriers, improvements to homework compliance and treatment engagement in underserved communities (34–36). Additionally, self-guided treatments require little or no therapist input, creating the opportunity for speech therapists to integrate it with their programs without requiring further training. The intensive nature of speech therapy can be a barrier to receiving the psychological support PWS often need (30), but a self-guided version of VRET offers greater flexibility. Given therapist interaction is often anxiety-inducing (37) and avoided (38), a self-guided option for VRET may also be more approachable.

Although trials of VRET for PWS are scarce, findings from several randomized trials support its use for reducing social anxiety in the general population (39–47). Most studies do not report their protocol’s theoretical basis to exposure, but at least two mention either following the emotional processing (45) or the inhibitory learning (41) approaches. Except for one early study (44), all trials to date have demonstrated either the superiority of VRET compared to waitlist control (41, 43, 45) or equivalence to *in vivo* exposure or CBT (40–42, 45, 47). Two studies also reported long-term maintenance of symptom reduction (39, 46). However, null findings regarding the reduction of fear of negative evaluation in three studies (40, 45, 47) suggest factors relating to the virtual nature of exercises may complicate VRET’s ability to target this key mechanism. Three meta-analyses provide further support for VRET (25–27). Findings from all three studies found that VRET was effective in reducing social anxiety symptoms and showed similar efficacy to other social anxiety treatments. This broad support from studies with robust methodologies provides strong support for the use of VRET in social anxiety.

Three randomized trials have assessed the efficacy of VRET targeting social anxiety using consumer technology and novel technological approaches. One of these studies used a regular consumer VR headset (48), whereas the other two used smartphone-based VR headsets (49, 50). The latter uses built-in smartphone capabilities allowing individuals to move their head around a virtual scene when the phone is inserted into a compatible headset. Whilst computational power remains lower than traditional headsets, this modality drastically widens access to VR. All three studies used 360° videos to create their virtual environments. These environments use a spherical video recording of a real environment simulating an anxiety-inducing situation. 360° video environments are much easier to create than computer-generated environments. They also result in improved perceived realism, which can support reductions in fear of negative evaluation, as fear-relevant stimuli such as faces are easier to register (50, 51). However, this modality offers less flexibility in terms of interaction with the virtual scene in comparison to computer-generated environments. One of these studies also replicated the therapist function virtually using automated voiceover prompts (48). Findings from all three studies showed superiority of VRET to waitlist control. These results are promising for the use of fast-developing consumer VR.

To date, only two studies have assessed the use of exposure therapy for reducing social anxiety amongst PWS, one using *in vivo* exposure (52) and the other using VRET (53). The *in vivo* study adopted a 10-session protocol targeting public speaking fears following the emotional processing rationale (52). Speech exercises involved both scripted speeches using stuttering-specific words and sounds, as well as spontaneous speech. A reduction in social anxiety symptoms was observed across participants, though data were not statistically analyzed. The VRET study used two exposure sessions which also targeted public speaking fears (53). However, it is unclear how these sessions followed either of the theoretical rationales for exposure. Additionally, the inclusion of a so-called ‘chill’ session, which participants could retreat to if feeling too anxious, could have had the inadvertent effect of reinforcing avoidant behaviors. In addition, no data or statistical analyses were reported, making it difficult to draw conclusions. Whilst both studies tailored treatment to stuttering, they were limited to targeting public speaking fears. Despite these methodological issues, these preliminary results provide a useful basis to evaluate further developments in stuttering-specific VRET.

A limitation for most of these trials is reliance on self-report measures of social anxiety. Neither of the exposure studies, nor the aforementioned trials of stuttering-specific CBT, measured changes in physiological arousal. Physiological activity is strongly linked to social anxiety (54–57), and there are several biomarkers of social anxiety that are specific to stuttering. These include lower resting heart rate variability (58), lower heart rate during anticipation (59), higher heart rate during speech task (60, 61), and higher skin conductance level during feared words (62). Physiological activity was only measured in one of the above randomized VRET trials (44). Findings showed a decrease in resting heart rate across the course of VRET. Another non-randomized trial found that HR in adolescents was reduced throughout a single VRET session, though this was not statistically analysed (63). However, in comparison to skin temperature and electrodermal activity, heart rate is less indicative of social anxiety (57). Future VRET trials should include physiological measures to comprehensively examine VRET’s efficacy. Choice of measures and experimental design should be informed by what best represents the expected shifts in social anxiety symptoms.

The aim of the current study is to conduct a randomized controlled pilot trial assessing the effectiveness of VRET, created using 360° video, for reducing social anxiety in PWS. The intervention is designed for PWS with both clinical and sub-clinical levels of social anxiety. As well as being the first randomized pilot to assess VRET in stuttering, it is the first test of VRET targeting social anxiety that is both remotely delivered using smartphone-based VR and assisted by a virtual therapist. Changes in social anxiety will be assessed both by self-report measures and physiological activity. Self-report measures will also be used to assess thoughts associated with stuttering, as well as stuttering characteristics. It is hypothesized that VRET will reduce social anxiety symptoms and the negative thoughts associated with stuttering. We also expect to observe physiological changes that indicate a reduction of social anxiety. This includes patterns of activity associated with social anxiety in stuttering. However, as VRET focuses entirely on social anxiety and explicitly instructs PWS not to focus on speech outcomes, no improvements to stuttering characteristics are expected.

## 2. Methods

### 2.1. Study design

**Figure 1:**
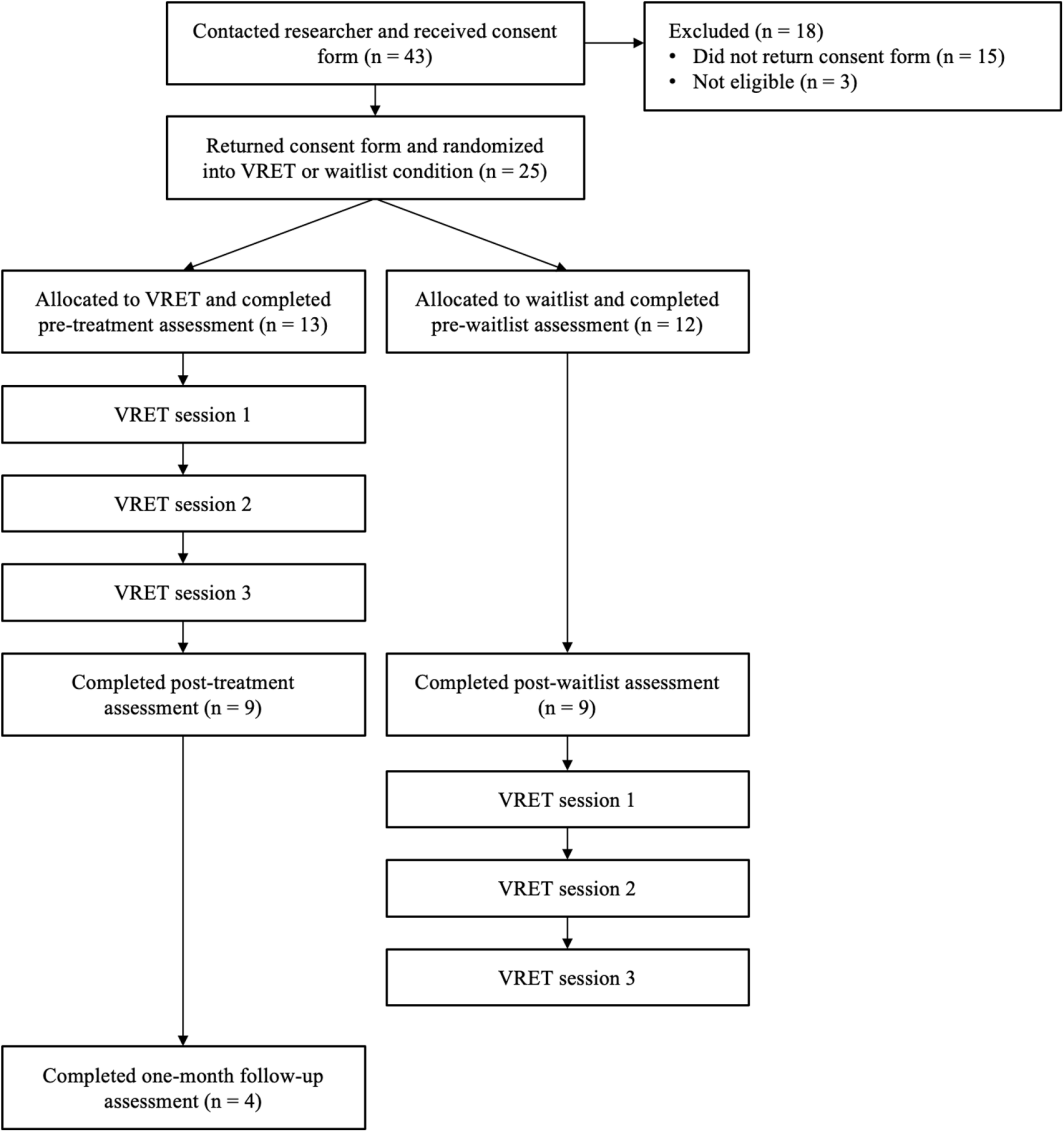
Flow diagram illustrating experiment procedure.

A parallel-group randomized controlled pilot trial was conducted, consisting of smartphone-based VRET and waitlist control conditions across a period of two months. Participants were randomized into the two conditions, and the same battery of measures assessed outcomes at pre- and post-treatment (or equivalent time points for waitlist participants at weeks one and three, respectively). Those in the VRET condition also completed the same battery of measures at a one-month follow-up. Waitlist participants received the same VRET intervention after the second measurement point. The study procedure is illustrated in Figure 1.

The pilot trial was approved by the Science Engineering Technology Research Ethics Committee at Imperial College London (reference number: 21IC7055). The report is written in line with the Consolidated Standards of Reporting Trials (CONSORT) Statement and the extension for reporting trials on psychological interventions (64).

### 2.2. Participants

25 participants (see Table 1 for demographic statistics) were recruited and randomized into the VRET (*n* = 13) and waitlist (*n* = 12) conditions (see Figure 1). The majority male sample reflects the gender split of PWS within the population (65). Recruitment finished at the end of the allotted study period, which ran between September 2021 – June 2022.

**Table 1:** Participant demographics.

Variable	Response	VRET	Waitlist
N		13	12
Age M (SD)		32 (9.44)	39 (16.86)
Gender	Male	9	7
	Female	2	4
	Did not say	2	1
Ethnicity	White British	6	7
	Other white	1	0
	Black African	2	3
	White and black Caribbean	0	1
	Indian	2	0
	Did not say	2	1
Education	A-levels	1	3
	Degree	9	6
	Other	1	2
	Did not say	2	1
Phone model	iPhone	10	6
	Android	3	6

Note: VRET, virtual reality exposure therapy.

Participants were recruited through online adverts on the British Stammering Association’s website (Stamma.org), and through local stuttering groups across the United Kingdom. Inclusion criteria were (1) Person who stutters, (2) English-speaking, (3) United Kingdom resident, (4) age over 18, (5) in possession of a smartphone (iPhone/Android) and headphones/earphones, (6) no previous participation in the feasibility trial or focus groups for same treatment, (7) no current experience of psychosis/schizophrenia/epilepsy/dementia/amnesia/bipolar disorder/intellectual disability/autism spectrum disorder, (8) no current use of tranquilizers or change in dosage of antidepressants in the past 6 weeks, (9) no current suicidal ideation, (10) no alcohol/substance dependence, (11) no severe cognitive impairment, (12) no treatment for social anxiety within the last year, (13) not participating in any other psychotherapeutic treatments during the study, and (14) no experience of severe cyber/motion-sickness. All participants who completed the pre- and post-treatment assessments (or equivalent for waitlist) received a £10 Amazon e-voucher.

### 2.3. Procedure

Individuals interested in participating who met the eligibility criteria were encouraged to contact the researcher *via* email. After expressing interest, prospective participants were sent the information sheet and consent form. Once the consent form was returned, participants were immediately randomized into either the treatment or waitlist condition. This was done by using a random number generator (random.org) to create a sequence of 0s and 1s. This sequence then determined condition allocation based on the order of returned consent form (0 = waitlist, 1 = treatment). Random sequence generation, participant enrolment, and random assignment to conditions was conducted by the primary researcher.

All participants were then asked to provide their home address to which cardboard VR headsets could be sent. Waitlist participants were not explicitly told that they had been allocated to the control condition but were notified that there would be a three-week delay until they could begin the treatment program and that there would be a series of assessments to complete in the meantime. Once the cardboard headset was delivered, participants were sent a link to complete the pre-treatment assessment. This consisted of four self-report questionnaires and a behavioral assessment task. The latter assessed participants’ distress in response to a speaking task. Participants were directed to a 360° YouTube video and instructed to watch it using their smartphone placed into the cardboard headset. The task involved participants giving a five-minute presentation to a panel of three people with the instructions to “discuss something that you recently achieved that you are proud of”. Participants had two minutes to prepare their speech whilst viewing an empty room, before the video showed the three panel members entering the room. Participants were encouraged to speak for as long as they could but were free to finish early. They were then re-directed back to the online assessment and asked to rate the level of distress experienced in the speech task. Participants were asked to notify the researcher when the assessment had been completed.

After this task, the treatment participants were given access to the first session of VRET, whilst the rest began the waitlist period. Three weeks later, once those receiving VRET had completed the treatment program, participants were sent a link to the post-treatment assessment. This followed the same procedure as the pre-treatment assessment. Participants were asked to inform the researcher when the assessment was complete and were then given the option to receive the Amazon e-voucher. Waitlist participants were also then given access to the first session of VRET. One month after completing the post-treatment assessment, participants in the treatment condition were sent a link to the same battery of assessments as part of the follow-up assessment. This followed the same procedure as before, and participants were asked to inform the researcher when it was complete.

Two participants in the treatment condition conducted the pre- and post-treatment assessments in-person at Imperial College London to measure physiological activity during the behavioral assessment task. All participants were offered this option, however United Kingdom Covid-19 restrictions hindered our efforts to recruit in-person participants during the trial period. These participants watched the same 360° video through an HTC Vive Pro headset. The same procedure was followed, but before the speech preparation and after the speech, participants watched an unrelated five-minute video on zoo animals to establish baseline physiological measures. The researcher left the room whilst the participants completed the behavioral assessment task.

### 2.4. Intervention

The current design of VRET was informed by a review of VRET literature (51), practical considerations (including Covid-19 restrictions), and input from PWS. The participatory design process involved focus groups with PWS (*n* = 5) and stuttering experts (*n* = 4) to gain insight on treatment design. The first of these was used to gather insights on the broad topic of tech-assisted mental healthcare, VRET, and relevance of these methods in stuttering. These reflections helped to inform the first iteration of VRET which was then evaluated in a small-scale feasibility trial to gain qualitative feedback on participants’ user experience. A second round of focus groups also gathered reflections on initial design features. Alterations were made based on this feedback, resulting in the final version of VRET evaluated in this pilot trial. Further details of the participatory design process will be published in a future article.

VRET was adapted for remote delivery so that the trial could be conducted despite ongoing Covid-19 restrictions. For that reason, a smartphone VR app developed in Unity and a cardboard smartphone-based VR headset (Virtual Real Store Google Cardboard V2) were used. The app uses the phone’s inertial sensors to determine head position within the 3D environment and modifies the visual and auditory rendering accordingly. Two images are displayed on the phone’s screen showing where the individual is looking in the scene. Both images are identical (monoscopic rendering) and are projected separately to each eye when the phone is inserted into the cardboard headset. Participants were instructed to use headphones for the audio delivery, which was spatialized only in the café environment (background noise). Mono audio in both ears was used for the rest of audio sources in the other scenarios. From the home page of the app, participants could access a page with a plant that tracked their progress by growing after each exercise. This was designed as a motivational tool to encourage participants to finish the course of treatment.

All participants completed VRET sessions remotely. Android and iOS versions of the app were created and downloaded through the Google Play Store and iOS App Store, respectively. The treatment program consisted of psychoeducation as well as exposure exercises. These were completed across three weekly sessions which participants could complete at their own pace, meaning they could leave the app and return throughout the week. Each week, participants were emailed a code which unlocked the next session’s exercises. All participants received codes to access all three sessions, unless they requested to stop the treatment early. The low number of sessions aimed to balance the amount of treatment content with fatigue and declining motivation that was expected from delivering the treatment remotely. In line with the remote delivery, participants were led by a virtual therapist, which took the form of pre-recorded 360° video clips.

The treatment program began with psychoeducation, consisting of the virtual therapist presenting information to the participant through a series of 360° video clips. These covered the mechanisms underlying social anxiety, principles of exposure therapy and inhibitory learning, treatment structure and how to use the app. This section was also used to help the participant formulate a goal for what they would like to achieve. A total of 17 clips (13 min) were presented and all had to be completed within the first session and before progressing to exposure.

Each treatment session consisted of three exposure exercises based on the same three social scenarios: ordering a drink at a café, telephone interaction, and public speaking (see Supplementary Appendix A for details). These exercises were created using 360° video clips. For the performative speech task, a single video clip displayed an audience in a room. For the interactive café and phone exercises, alternating clips of people talking and being silent were used to facilitate turn-taking. Participants indicated the end of their turn by clicking a button to play the next clip.

Exercises were adapted to target the specific stimuli associated with social anxiety in stuttering. Each exercise was different and varied in difficulty, but this was randomized across sessions. Exposure followed the inhibitory learning (21) approach, by aiming to develop new non-threatening associations with feared stimuli that can inhibit previous fearful associations. This involved a technique called expectancy violation, which aimed to disprove beliefs and expectations that the participant held about the outcomes in each scenario. Before each exercise, the virtual therapist guided participants’ preparation by helping them define their expectations and the safety behaviors they might perform. This step was also tailored by offering examples of expectations and safety behaviors that PWS often experience. After each exercise attempt, the virtual therapist helped participants evaluate how the scenario compared to their expectations. The aim was not to reduce anxiety during exercises as habituation techniques would recommend (20), but to increase understanding that feared expectations are overestimated, and to reinforce new associations to the point where anxiety is manageable. Participants were advised to finish each exercise when they believed they had made progress towards these aims. Sessions were designed to last approximately 20–30 min, but this was not measured.

Given that PWS are at greater risk of reacquisition of fear from continued reactions to their speech, the intervention also had a particular emphasis on preventing it. In line with inhibitory learning techniques, several of the exposure exercises included audience reactions that align with existing expectations (e.g., negative facial reactions of audience in public speaking exercise). This was used to promote learning to deal with negative outcomes (22).

### 2.5. Measures

#### 2.5.1. Self-report measures

All self-report measures were assessed at pre-treatment, post-treatment (equivalent assessments for waitlist participants) and at one-month follow-up. All variables were created using mean scores.

##### 2.5.1.1. Social anxiety

Social anxiety symptom severity was assessed using the Social Phobia Scale (SPS) (66). SPS is a 20-item self-report questionnaire using a 5-point Likert scale, designed to assess performance and scrutiny fears. SPS has shown good psychometric properties in previous research (54). Cronbach’s *α* values for the main and follow-up sample were .96 and .79, respectively.

Fear of being negatively evaluated by others was assessed using the brief version of the Fear of Negative Evaluation scale (FNE-B) (67). FNE-B is a 12-item scale using a 5-point Likert scale and has demonstrated good psychometric properties (68). Cronbach’s *α* values for the main and follow-up sample were. 94 and .98, respectively.

##### 2.5.1.2. Thoughts about stuttering

The brief version of the Unhelpful Thoughts and Beliefs About Stuttering scale (UTBAS-6) (69) was used to assess negative thoughts commonly experienced by PWS. The scale is representative of thoughts experienced by PWS seeking treatment for social anxiety. UTBAS-6 is divided into three sub-scales assessing the frequency of thoughts, how much the individual believes them, and how anxiety-inducing they are. The same 6 thoughts are assessed across these sub-scales using a 5-point Likert scale. UTBAS-6 has shown good psychometric properties (69). Cronbach’s *α* values for the main and follow-up sample were. 97 and .87, respectively.

##### 2.5.1.3. Stuttering characteristics

The Wright and Ayre Stuttering Self-Rating Profile (WASSP) (70) was used to assess characteristics associated with stuttering severity. WASSP was developed under the rationale that stuttering treatment gains cannot be measured by fluency alone, and therefore assesses five factors associated with stuttering severity: behaviors, thoughts, affect, avoidance, and disadvantage due to stuttering. The scale is divided into five sub-scales reflecting these factors and contains 24 items using a 7-point Likert scale. Previous studies have shown WASSP to have good psychometric properties (71). Cronbach’s *α* values for the main and follow-up sample were.96 and.92, respectively.

##### 2.5.1.4. Speech-related distress

Real-time distress during the speech task in the behavioral assessment task was assessed using the Subjective Units of Distress Scale (SUDS) (72). This scale asks the individual to rate their level of distress during a task using a fear thermometer translating to an 11-point Likert scale. It has been used to assess state-level anxiety in several VRET trials (39, 40, 44, 47, 48).

#### 2.5.2. Physiological measurement

Physiological recording was conducted for two participants completing the behavioral assessment task in-person at pre- and post-treatment assessments. An Empatica E4 wristband was used to measure skin temperature, electrodermal activity (EDA), Heart rate (HR) and heart rate variability (HRV) during the speech task. A continuous recording was conducted across the four segments (pre-speech baseline, speech planning/anticipation period, speech task and post-speech baseline), and then split using a MATLAB script.

Root mean square of successive differences (RMSSD) and high frequency (HF) activity were used as measures of HRV. RMSSD and HF reflect the most suitable measures of HRV in the time and frequency domain, respectively (73), and both represent parasympathetic nervous system activity. ARTiiFACT (74) was used to process raw HR data and obtain HRV data for each segment.

Skin conductance level (SCL) and skin conductance response (SCR) were recorded to obtain both tonic and phasic facets of EDA, respectively. Number of SCR peaks per minute were also recorded for each segment. Ledalab (75) was used to process raw signals and obtain EDA data.

### 2.6. Statistical analyses

Multilevel models were used to analyze outcome data between pre- and post-treatment. This allowed for examination of main effects as well as exploration of the extent of random by-subject variation. Due to the low sample size and high level of attrition between post-treatment and follow-up, statistical analyses were not conducted to assess the change in outcomes between these points. Similarly, as only two participants provided physiological data, no statistical analyses were conducted on this data. Thus, for follow-up and physiological measures, we only report descriptive data.

Multilevel models use Maximum Likelihood estimation to minimize bias because of missing data, and data was analyzed on an intention-to-treat basis. Missing data was assumed to be missing at random, and Little’s test returned a non-significant result (*χ*^2^ = 24.59, df = 26, *p* = 0.54) to support this.

Multilevel models were created in R using the lme4 package (76). Models included an intercept and fixed parameters for the main effects of condition (treatment vs. waitlist), time (pre- vs. post-treatment) and their interaction. Deviation coding was used for these categorical variables (Waitlist = −0.5, Treatment = 0.5; Pre-treatment = −0.5, Post-treatment = 0.5). The fixed effect parameters can be interpreted in the same way as a traditional linear regression model that uses deviation coding. By-subject random intercepts for time were included, representing the amount of residual error accounted for by allowing intercepts, and thereby regression lines, to vary by subject. However, there were not enough observations to allow the time coefficient to vary by subject as the number of observations per participant must exceed the number of random effects.

For each model, age and gender were added as covariates. This only improved model fit for the model assessing changes to fear of negative evaluation [*χ*^2^ (2) = 9.02, *p* = 0.01], thus output reflects inclusion of these covariates. All measures were transformed using Box-Cox transformations after not meeting at least one assumption for parametric analyses (normality, skewedness, kurtosis, homogeneity of variance and extreme values). Analyses run on raw and transformed data revealed similar patterns in results, therefore raw data was used for final reporting. Multilevel models also met the assumptions of linearity and normality of residuals and random intercepts.

The lmerTest package (77) was used to perform hypothesis testing on speech distress, social anxiety, fear of negative evaluation and stuttering-related thoughts. This uses Satterthwaite’s method to estimate degrees of freedom and Type III ANOVA tables necessary to produce associated *p*-values for each fixed effect. For pre- to post-treatment data, Cohen’s d adapted for pretest-posttest-control designs (78) was used for assessing effect sizes, whilst follow-up data used Hedge’s g for repeated measures. The lmerTest package was also used to calculate the significance of adding random intercepts to each model by assessing model fit changes *via* a likelihood ratio test. This tests the null hypothesis that regression slopes do not vary significantly between participants. The Intraclass Correlation Coefficient (ICC) also assessed the proportion of variance in outcome data attributable to individual differences between subjects. The r2mlm package (79) was used to produce R-squared values to assess model fit. Separate R-squared values were produced indicating the variance accounted for by fixed effects, by-subject random intercepts, and the overall model.

To understand more about the effect of treatment, we also conducted equivalence tests on all measures. This provided extra information to the previous tests by effectively checking whether any fixed parameters resulted in a significant null effect. For stuttering characteristics, this was the only test conducted as no effect of treatment was expected. Equivalence tests were conducted in R using the “equivalence_test” function within the bayestestR package (80). Using the Bayesian method, it outputs a confidence interval for each fixed parameter, and an equivalence region which defines a set of parameter values that can be considered equivalent to zero. If the confidence interval lies entirely within this equivalence region, one can reject the null hypothesis of non-equivalence and assume no effect for that fixed parameter. The null hypothesis of non-equivalence is accepted if the confidence interval and equivalence region do not overlap. Any other outcome suggests the data is insufficient to draw conclusions about hypotheses.

We were unable to conduct an accurate *a priori* power analysis due to the lack of available reported fixed and random parameters to base our estimations on. Instead, sensitivity analyses were conducted after data was collected and analyzed, to determine the minimum treatment effect size required to achieve a sufficient level of power with the same sample size that we had. Using the SIMR package in R (81), we calculated the observed power for our multilevel models assessing treatment effects on speech-related distress, social anxiety, fear of negative evaluation and thoughts about stuttering. By varying the fixed parameter for the interaction between condition and time, we altered the observed power. We report the interaction coefficient that resulted in 80% power.

## 3. Results

Descriptive data for all self-report measures can be found in Table 2. Descriptive data for physiological data can be found in Table 3. The raw data for this pilot trial have been published in an open-access repository (82). Supplementary Appendix B comprises full details on multilevel model outcomes, random effects, and model fit.

**Table 2:** Descriptive statistics for self-report measures.

		Pre-treatment (*n* = 25)		Post-treatment (*n* = 18)		Follow-up (*n* = 4)	
Measure	Condition	M	SD	M	SD	M	SD
SUDS	Treatment	4.64	2.01	3.56	2.07	1.75	0.96
	Waitlist	4.44	2.40	5.63	4.00		
SPS	Treatment	0.72	0.47	0.57	0.36	0.38	0.18
	Waitlist	1.58	0.78	1.69	1.05		
FNE-B	Treatment	3.22	0.74	3.25	1.00	2.96	0.87
	Waitlist	3.67	0.85	3.78	1.05		
UTBAS	Treatment	2.36	0.75	2.39	0.74	2.18	0.36
	Waitlist	3.17	0.98	2.98	1.12		
WASSP	Treatment	3.87	0.91	3.66	1.05	3.50	0.67
	Waitlist	4.39	1.31	4.32	1.52		

Note: SUDS, subjective units of distress scale; SPS, social phobia scale; FNE-B, brief version of the fear of negative evaluation scale; UTBAS, unhelpful thoughts and beliefs about stuttering scale; WASSP, Wright and Ayre stuttering self-rating profile.

**Table 3:** Descriptive statistics for physiological data.

		Pre-treatment M (SD)				Post-treatment M (SD)			
Measure	Participant number	Pre-speech baseline	Anticipation	Speech task	Post-speech baseline	Pre-speech baseline	Anticipation	Speech task	Post- speech baseline
Skin temp. (°C)	1	34.00	34.38	34.15	34.18	31.38	31.30	31.19	31.00
	2	34.14	34.34	34.12	34.92	32.78	32.91	32.99	33.23
SCL (microS)	1	0.06 (0.02)	0.01 (0.03)	0.52 (0.21)	0.60 (0.13)	0.08 (0.06)	0.03 (0.06)	0.70 (0.12)	0.73 (0.11)
	2	0.16 (0.09)	0.10 (0.05)	0.13 (0.09)	0.71 (0.06)	0.14 (0.09)	0.29 (0.03)	0.35 (0.05)	0.47 (0.05)
SCR amplitude (microS)	1	0.09 (0.00)	0.03 (0.00)	0.04 (0.02)	0.12 (0.11)	0.04 (0.05)	0.03 (0.01)	0.03 (0.04)	0.03 (0.02)
	2	0.03 (0.01)	0.03 (0.01)	0.03 (0.01)	0.03 (0.01)	0.05 (0.03)	0.06 (0.03)	0.06 (0.02)	0.06 (0.03)
HR (bpm)	1	78.17	73.90	78.52	73.29	69.16	66.74	71.87	63.79
	2	53.99	60.26	62.27	56.22	60.32	62.98	61.95	58.58
RMSSD (ms)	1	37.26	36.55	65.91	49.12	39.76	48.91	76.01	50.64
	2	31.45	43.88	96.02	39.31	34.74	56.11	48.41	49.55
HF (ms^2^)	1	596.53	395.68	1823.79	926.75	323.55	497.90	1873.42	872.38
	2	224.40	475.12	560.05	430.28	318.22	839.68	968.36	732.95

Note: SCL, skin conductance level; SCR, skin conductance response; HR, heart rate; RMSSD, root mean square of successive differences; HF, high frequency.

### 3.1. Participant attrition

Four participants in the treatment group did not complete the post-treatment assessment. Welch’s t-test indicated no significant difference in pre-treatment SPS between participants who did (*M* = 0.72, *SD* = 0.54) and did not (*M* = 0.73, *SD* = 0.36) complete the post-treatment assessment, *t*(8.70) = 0.00, *p* = 0.99. Three participants in the waitlist group did not complete the equivalent assessment, with Welch’s t-test indicating no significant difference in pre-waitlist SPS between participants who did (*M* = 1.57, *SD* = 0.92) and did not (*M* = 1.62, *SD* = 0.03) complete the post-waitlist assessment, *t*(8.05) = 0.03, *p* = 0.87.

Of the nine participants in the treatment condition who completed the post-treatment assessment, four completed the follow-up assessment. Welch’s t-test found no significant difference in post-treatment SPS between participants who did (*M* = 0.54, *SD* = 0.19) and did not (*M* = 0.59, *SD* = 0.48) complete the follow-up assessment, *t*(5.43) = 0.05, *p* = 0.83.

### 3.2. Behavioral assessment task

The overall effect size revealed a large group difference in the change of speech task distress between pre- and post-treatment (d = −0.99). Distress scores decreased and increased for treatment and waitlist participants, respectively, but this difference was not significant, b = −1.59; *F*(1, 16.86) = 2.53, *p* = 0.13. The equivalence test found that the confidence interval for the Condition*Time interaction parameter [CI (-3.29, 0.11)] and equivalence region (*Δ*_L_ = −0.26; *Δ*_U_ = 0.26) partially overlapped. Therefore, we are not able to draw conclusions about the effect of VRET on distress. The sensitivity analysis revealed that the coefficient for this interaction would have had to equal −2.40 to achieve 80% power. As the observed effect (–1.59) was smaller, this suggests that the current study was not sensitive enough to detect the effect of treatment. Overall model fit was high (*R*^2^ = 0.70) and was significantly improved by including by-subject random intercepts, *χ*^2^ (1) = 9.34, *p* = 0.002; ICC = 0.68. Speech-related distress was also considerably lower at one-month follow-up compared to post-treatment (g = −0.65). These findings suggest that our VRET intervention had a sizable effect on changes to speech-related distress in the current sample, though this was not significant. However, follow-up findings indicate that distress continued to decline post-treatment.

### 3.3. Physiological response

Skin temperature remained relatively stable across segments for both participants, but there was a noticeable overall decrease from pre- to post-treatment. Skin conductance level (SCL) increased in the speech task compared to the anticipation period for both participants at both pre- and post-treatment. However, SCL was noticeably higher at post-treatment for the speech task. SCL also continued to increase in the post-speech baseline for both participants at both measurement points. Comparatively, mean skin conductance response (SCR) amplitude remained relatively stable across segments for both participants at both measurement points, and there was little difference between pre- and post-treatment.

For participant 1, across both measurement points, heart rate was raised for both the pre-speech baseline and speech task but was lower during anticipation. For participant 2, heart rate was raised during anticipation and the speech task across both measurement points. For both participants, there was a slight decrease in heart rate during the speech task between pre- and post-treatment. However, heart rate during anticipation decreased between pre- and post-treatment for participant 1, whilst participant 2 showed the opposite effect.

At pre-treatment, root mean square of successive differences (RMSSD) was shown to peak during the speech task for both participants. A similar pattern was shown for participant 1 at post-treatment, though RMSSD peaked during anticipation for participant 2. RMSSD during the speech task increased between pre- and post-treatment for participant 1 but decreased for participant 2. However, RMSSD during pre-speech baseline increased for both participants. High frequency (HF) activity was raised during anticipation and peaked during the speech task for both participants across both measurement points, but this peak was far more substantial for participant 1. For participant 1, the peak was similar between pre- and post-treatments, but it increased for participant 2. Pre-speech baseline HF changes were also different between participants. These findings suggest VRET had mixed effects on physiological changes for the two participants. Nevertheless, they also indicate the capacity of VRET to evoke physiological response.

### 3.4. Social anxiety symptoms

Social anxiety marginally decreased from pre- to post-treatment in the treatment group whilst increasing for waitlist participants (d = −0.41), though the difference was not significant b = −0.28; *F*(1, 19.56) = 3.10, *p* = 0.09. The equivalence test found that the confidence interval for the Condition*Time interaction parameter [CI (–0.61, 0.04)] and equivalence region (*Δ*_L_ = −0.08; *Δ*_U_ = 0.08) partially overlapped. Therefore, there is insufficient evidence to support the predicted reduction in social anxiety from VRET. The sensitivity analysis revealed that the coefficient for this interaction would have had to equal −0.40 to achieve 80% power. As the observed effect (–0.28) was somewhat smaller, this suggests that the current study was not sensitive enough to detect the effect of treatment. However, waitlist participants scored significantly higher overall, b = −1.00; *F*(1, 26.08) = 15.91, *p* < 0.001. Overall model fit was high (*R*^2^ = 0.91) and was significantly improved by including by-subject random intercepts *χ*^2^ (1) = 28.01, *p* < 0.001; ICC = 0.89. In addition, social anxiety was lower at one-month follow-up compared to post-treatment (g = −0.34). These findings failed to show that VRET was more effective than waitlist at reducing social anxiety, but indicate that VRET may be able to affect social anxiety longer-term.

### 3.5. Fear of negative evaluation

There was little change in the fear of negative evaluation for both treatment and waitlist conditions (d = −0.09). The multilevel model revealed no significant difference between conditions regarding the pre-post change in scores, b = −0.29; *F*(1, 18.37) = 1.81, *p* = 0.19). The equivalence test found that the confidence interval for the Condition*Time interaction parameter [CI (-0.64, 0.43)] fully contained the equivalence region (*Δ*_L_ = −0.09; *Δ*_U_ = 0.09). From these tests we are not able to draw conclusions about VRET’s effect on fear of negative evaluation. The sensitivity analysis revealed that the coefficient for this interaction would have had to equal −0.65 to achieve 80% power. As the observed effect (–0.29) was smaller, this suggests that the current study was not sensitive enough to detect the effect of treatment. However, women scored significantly higher overall, b = −0.81; *F* (1, 22.86) = 5.73, *p* = 0.03. Overall model fit was high (*R*^2^ = 0.88) and was significantly improved by including by-subject random intercepts, *χ*^2^ (1) = 19.66, *p* < 0.001; ICC = 0.78. Fear of negative evaluation was also somewhat lower at one-month follow-up compared to post-treatment (g = −0.28). These findings preclude conclusions about VRET’s ability to reduce fear of negative evaluation, but indicate that VRET may be able to affect it longer-term.

### 3.6. Thoughts and beliefs about stuttering

There was little change in stuttering-related thoughts for treatment participants, with a slight decline for those in the waitlist group (d = 0.25). Nevertheless, the difference between conditions regarding the pre-post change was not statistically significant, b = 0.15; *F* (1, 20.53) = 0.30, *p* = 0.59. The equivalence test found that the confidence interval for the Condition*Time interaction parameter [CI (–0.40, 0.71)] fully contained the equivalence region (*Δ*_L_ = −0.09; *Δ*_U_ = 0.09). These test results reveal that the current data is insufficient to draw conclusions about the effect of VRET on stuttering-related thoughts and beliefs. The sensitivity analysis revealed that the coefficient for this interaction would have had to equal 0.66 to achieve 80% power. As the observed effect (0.15) was smaller, this suggests that the current study was not sensitive enough to detect the effect of treatment. However, waitlist participants scored significantly higher overall, b = −0.73; *F*(1, 26.43) = 5.19, *p* = 0.03. Overall model fit was high (*R*^2^ = 0.79) and was significantly improved by including by-subject random intercepts, *χ*^2^ (1) = 17.39, *p* < 0.001; ICC = 0.78. In addition, level of stuttering-related thoughts was similar between post-treatment and one-month follow-up (g = −0.20). Thus, our findings failed to demonstrate a reduction in stuttering-related thoughts and beliefs from VRET as expected.

### 3.7. Stuttering characteristics

There was a small decrease in self-reported stuttering between pre- and post-treatment for the VRET group, whilst the waitlist group remained relatively stable (d = −0.12). The confidence interval for the Condition*Time interaction parameter [b = −0.38; CI (-0.94, 0.18)] fully contained the equivalence region (*Δ*_L_ = −0.12; *Δ*_U_ = 0.12). Therefore, we are not able to draw conclusions about this effect. Overall model fit was high (*R*^2^ = 0.87) and was significantly improved by including by-subject random intercepts, *χ*^2^ (1) = 26.32, *p* < 0.001; ICC = 0.82. In addition, self-reported stuttering was similar between post-treatment and one-month follow-up (g = −0.10). From these findings, we are not able to draw conclusions on whether VRET affected stuttering characteristics.

## 4. Discussion

The current pilot trial investigated the efficacy of remotely delivered smartphone-based VRET designed specifically to reduce social anxiety associated with stuttering. Findings showed that while social anxiety did decrease in VRET participants, this was not significantly different relative to the waitlist control group. Similar results were found for fear of negative evaluation and state-level distress reported during the behavioral assessment task. However, there were encouraging findings indicating the potential of VRET to decrease social anxiety longer-term. Nevertheless, this effect is based on a small sample and was not statistically analyzed, meaning it is not entirely conclusive.

A mixed picture emerged regarding how VRET affected physiological outcomes. The overall decrease in skin temperature is promising, as this is a reliable measure of subclinical social anxiety (57). The decline in heart rate during the speech task across both participants is also encouraging given its link to speech-related social anxiety (60, 61). However, the increase in skin conductance level during the speech task was unexpected as it represents heightened stuttering-related anxiety (62). Resting heart rate variability and heart rate during anticipation are also suggested indicators of stuttering-related anxiety (58, 59), yet there were no observable patterns for these measures. We cannot draw any conclusions from these pilot findings given the small sample size, however. Thus, we cannot infer these observed trends would extrapolate onto the wider population. However, this is the first randomized pilot trial of VRET to include physiological measurement, and further research is required to assess these effects more rigorously.

Findings failed to demonstrate the superiority of our VRET protocol over waitlist at reducing social anxiety amongst PWS. This contradicts early promising research which suggests exposure therapy may be an effective technique for this group (52, 53). Nevertheless, the current pilot has overcome several methodological concerns from prior studies to provide the most rigorous assessment yet for the use of VRET in stuttering. Our results are also at odds with most research supporting VRET’s use for reducing social anxiety amongst the non-stuttering population (40–43, 45, 47). Only one previous randomized trial failed to show a significant reduction of social anxiety from VRET (44). Nevertheless, findings from the sensitivity analysis revealed that, with the current sample size, the observed treatment effect was not sufficient for achieving necessary power. It is likely that the small sample size contributed to this as the medium-sized treatment effect was nearing the necessary effect size for achieving sufficient power. As a result, our findings preclude conclusions about the effectiveness of VRET at reducing social anxiety amongst PWS. Future trials of VRET for stuttering should rely on larger sample sizes to ensure studies are sufficiently powered.

It is also likely that the treatment effect in the current study was impacted by the observed group difference in social anxiety. This saw symptom severity more than halved in the treatment group at both pre- and post-treatment. Severity of stuttering-related thoughts was also found to be significantly higher in the waitlist group. It is plausible that the small sample size failed to ensure an equal dispersion of scores across groups, despite randomization. The larger proportion of women in the waitlist group may underlie this difference, as women are more likely to develop heightened social anxiety (83). As a result, the potential for further decreases to social anxiety may have been more limited in the treatment group, causing a floor effect for this test of VRET against waitlist. Therefore, the lack of treatment effect might represent a Type II error, and interpretation of findings should be done with caution. This further illustrates the need for future trials to recruit larger samples, to ensure randomization results in more equal groups.

Our findings also showed that social anxiety and speech-related distress were lower at one-month follow-up compared to post-treatment. This result is not directly in line with previous studies, which found no further reductions in social anxiety, but long-term maintenance of treatment gains at six months to six years post-treatment (39, 41, 46). We cannot make conclusive inferences based on these findings due to the small sample size. However, the observed pattern is promising for VRET’s ability to support longer-term learning beyond the end of treatment. In particular, inhibitory learning techniques that were adopted in exposure exercises may be well suited to supporting broader learning strategies that can be applied after treatment finishes. In addition to recruiting larger sample sizes, further trials would benefit from comparing VRET against a control or comparison group to provide a more conclusive assessment of its long-term effects amongst PWS.

The lack of idiosyncrasy within VRET sessions may have contributed to the lack of effects between pre- and post-treatment. Other trials have successfully tailored social anxiety treatments to PWS but had greater flexibility in personalizing sessions and were not restricted by pre-made exercises (14, 17, 18). Despite the inclusion of stuttering-specific features, a common theme reported by participants was that exercises were not relevant to their personal experience. One participant mentioned that the therapist exchange to discuss personal emotions and experiences was not sufficiently recreated by the virtual therapist to the detriment of treatment engagement. Another participant mentioned that exercises did not recreate scenarios and stimuli that made them anxious, causing them to drop out of the study. Significant random effects also suggest substantial variation across participants as expected. The current design attempted to personalize sessions, for example by instructing participants to use their feared words in the public-speaking exercise. However, it is unlikely that the broader nature of the three scenarios were relevant to all participants equally. Future VRET design work should include a wider variety of exposure scenarios and continue to include PWS in the participatory design process.

The current pilot trial was unable to demonstrate the effectiveness of remotely delivered VRET designed specifically for PWS. Research in self-guided VRET for social anxiety is still in its infancy, but our results are not aligned with previous studies supporting virtual therapists (48) and smartphone-based VR (49, 50). However, trials of automated CBT for PWS have also had mixed success in reducing social anxiety (17, 18). In the current VRET protocol, participants may have struggled to develop a supportive and collaborative relationship with the virtual therapist, which is an important factor for successful speech therapy and psychological treatment in PWS (30, 84). The virtual therapist may have also struggled to facilitate inhibitory learning, given there was limited assistance if participants struggled. A comparison of therapist-led vs. fully remote delivery is required to uncover the effects of treatment format on therapeutic outcomes.

Feedback from participants suggests that issues with equipment and VRET design may have inhibited the effectiveness of novel treatment techniques. Several participants reported discomfort and a lack of visual immersion from the cardboard headsets. These headsets have the benefit of being cheap and compatible with any smartphone but are far less immersive than the Samsung Gear VR headset used in the aforementioned trials of smartphone-based VRET (49, 50). Importantly, they were chosen as they could be posted to participants, thereby ensuring our trial could take place despite United Kingdom Covid-19 restrictions at the time. Their use might have particularly impacted fear of negative evaluation, which did not change between pre- and post-treatment. This finding was less surprising given several VRET trials have reported similar effects (40, 45, 47). However, design limitations suggested to have caused these results were addressed in the current trial. In particular, one-to-one interaction and 360° video was used in an attempt to ensure facial feedback could be registered more easily (51). Despite this, the cardboard headset may have prevented faces from being registered by participants. Realistic interaction may have also suffered by using 360° video, as turn-taking relied on participants clicking buttons to play subsequent videos. Additionally, some videos could take a few seconds to load, and were not perfectly aligned with other videos. Thus, further work is required to understand if there are limits to using smartphone-based VRET and 360° video.

The remote nature of treatment might have also hampered participant’s motivation (85, 86). Only nine of the thirteen participants in the treatment condition completed the course of treatment, and several participants required prompting to complete sessions. The attrition rate was much lower in previous VRET trials using a virtual therapist (48) and smartphone-based VR (49, 50), but these were conducted in-person. Engagement may have been strengthened by having appointments to attend. In our pilot trial, other factors, such as choice of scenarios and headset discomfort, might have frustrated participants and contributed to disengagement. The motivational features built into treatment, including the virtual therapist and plant, may not have been sufficient to overcome disengagement. Thus, although self-guided VRET holds potential for PWS, further research needs to address factors associated with disengagement when treatment is conducted remotely. Future studies should also be conducted remotely to fully simulate the context in which VRET is used.

Our results also failed to show an effect of VRET on thoughts about stuttering that are related to social anxiety. This is consistent with our primary findings which suggest that participants who received VRET failed to learn new associations, and in line with previous trials suggesting CBT does not improve learning when integrated into speech therapy (14, 18). Speech therapy may be sufficient for targeting stuttering-related thoughts, whilst VRET and other social anxiety treatments may offer limited additional benefits. Similarly, findings showed VRET had little effect on speech outcomes, though we were not able draw conclusions about this effect from the equivalence test result. This observed pattern is in line with previous results suggesting limited benefits to stuttering from standalone CBT (17). Further research should examine whether VRET can aid speech outcomes when integrated with speech therapy, as has been shown for CBT (18).

### 4.1. Limitations

There are several limitations to our pilot trial. First, the sample size was relatively small, and sensitivity analyses revealed that a larger effect would have been necessary on all measures to achieve a sufficient level of power with the current sample size. Participant recruitment was a particular challenge given PWS only represent 1% of the general population (87) and social anxiety does not affect all PWS. Additionally, there was a large amount of variance in participants’ responses. This is supported by the finding of significant random effects. The current study did not exclude participants based on anxiety level, which might have increased variability. This may have also contributed to several models showing low *R*^2^ values for fixed effects, suggesting poor model fit. Together, these factors may help to explain why our results for speech task distress and social anxiety failed to reach significance, despite showing moderate to large effect sizes. However, as the observed treatment effect size was smaller compared to other similar studies (45), this will have also decreased power. Thus, caution should be taken in interpreting effects.

Second, a lack of proper allocation concealment may have introduced selection bias. Because the primary researcher had knowledge of participant characteristics and allocation sequence, they may have been biased when allocating participants to conditions based on the random sequence. As the trial was conducted remotely, there were limited options for concealing this allocation from the primary researcher, however.

Lastly, there were issues with participant disengagement and attrition. There are several factors that may have contributed to this, including headset discomfort and lack of personalized sessions. Several of these design choices were made so that the study could be conducted remotely due to the United Kingdom Covid-19 restrictions at the time (March 2020 – July 2021). Three consecutive lockdowns made study planning and participant engagement very challenging. Conducting the pilot trial remotely also meant that it was less controlled, as researchers were not able to ensure that procedures were followed properly. Although multilevel models with maximum likelihood estimation were used to deal with missing data, participant attrition has likely biased our findings.

### 4.2. Strengths

Despite its limitations, this pilot trial has several strengths. First, the design of VRET followed a methodical process that focused on integrating insights from PWS *via* participatory design. This provided nuanced guidance in ways literature could not, whilst user testing helped with understanding which features were more or less appropriate for PWS. Focus group insights were combined with expert opinions and psychological literature to formulate a holistic and practical protocol. Other researchers have emphasized the importance of the participatory design process and its adoption for VRET (88). However, to our knowledge, this is the only randomized pilot trial of VRET that was developed using this process. Future VRET designs should continue to involve target groups to increase accessibility and ensure maximum relevance to specific fears. Second, this is the only study to use robust methods to examine VRET’s efficacy in stuttering. We compared VRET against a control group, and analyzed results with appropriate statistical analyses. Lastly, despite limitations of remote experiments, the current study was able to involve a variety of people from across the United Kingdom. This demonstrates the possibilities of using such treatments to remove barriers, including those related to travel and geography. In sum, the current study contributes to the literature investigating stuttering-specific social anxiety treatments and provides the most robust assessment yet for VRET in stuttering.

## 5. Conclusion

The complex nature of comorbid stuttering and social anxiety requires careful consideration regarding practical treatment implementation. VRET is an appealing method for achieving further integration of speech and anxiety treatments, and our pilot results provide an early insight into what methods might be appropriate. Nevertheless, further work to perfect VRET design is required to maximize effectiveness and appropriateness in the lives of PWS. Our outcomes also pave the way for further design improvements and future research to better understand how VRET can play a role in mental health treatment for PWS.

## Data Availability

The datasets presented in this study can be found in online repositories. The names of the repository/repositories and accession number(s) can be found below; http://osf.io/p3ahn.
