# Supplementary material for: Virtual reality exposure therapy for reducing social anxiety in stuttering: A randomized controlled pilot trial
Source: Front Digit Health. 2023 Feb 9;5:1061323. doi: 10.3389/fdgth.2023.1061323 (PMC9947508; doi:10.3389/fdgth.2023.1061323)
Supplement: Supplementary file 1 [file Table1.docx]

Supplementary Material

Appendix A. VRET exercises

| **Session no.** | **Café** | **Telephone** | **Public speaking** |
| --- | --- | --- | --- |
| 1 | Participant must order a drink from a café – they can order whatever they like, and the server will ask if they want anything else.  Specific features: participant has to say their name, immersive café audio. | Participant makes a phone call to the local water company about a leak which was fixed but is now broken again. Participant must liaise with the caller to organise another appointment to fix the leak.  Specific features: someone else in the room (not directly observing), participant must give their name, participant has to be assertive when caller doubts whether original leak wasn’t fixed properly. | Participant must give a two-minute speech to an audience of two people on the topic of “is it suitable to keep pets in the city?”  Specific features: countdown clock. |
| 2 | Participant must order a drink from a café – they must choose between tea and coffee, and assert themselves when server is incorrect when repeating their order. A different server also gives them the wrong drink and does not accept that it is incorrect before the original server steps in.  Specific features: participant has to say their name and then spell it when asked by server, participant must assert themselves when server is incorrect when repeating their order, participant must also assert themselves when the different server gives them the wrong drink and refuses to accept this is incorrect, immersive café audio. | Participant receives a phone call from a friend called Mike whose party they were at the night before – Mike has found their wallet and wants to return it. Participant has a brief discussion about when they can come over to return it.  Specific features: participant has to answer the ringing phone. | Participant must give a two-minute speech to an audience of four people on the topic of “How do we fight climate change?”  Specific features: participant is asked to use words that often cause them to stammer, more controversial topic, countdown clock. |
| 3 | Participant must order a drink from a café – they must choose between tea and coffee, and assert themselves when server is incorrect when repeating their order. A different server also gives them the wrong drink but apologises before original server gives them the correct drink.  Specific features: participant has to say their name and then spell it when asked by server, participant must assert themselves when server is incorrect when repeating their order, participant must also assert themselves when the different server gives them the wrong drink, immersive café audio. | The participant must make a business phone call – they work for a record label and must convince an investor to invest in a DJ they represent. The investor is not initially convinced and needs persuasion. Participant must liaise with investor to organise another meeting to discuss further.  Specific features: Two other colleagues in the room observing participant, colleague is slightly hostile. | Participant must give a two-minute speech to an audience of eight people on the topic of “Will Brexit be successful for the UK?”  Specific features: participant is asked to use words that often cause them to stammer, some audience members are bored/react negatively, more controversial topic, countdown clock. |

Appendix B. Supplementary tables

Supplementary table 1 – Multilevel model parameters, associated p-values and effect sizes (Cohen’s d/Hedge’s g)

|  |  | **Pre- vs. Post-treatment** | | | | **Post-treatment vs. follow-up** |
| --- | --- | --- | --- | --- | --- | --- |
| **Measure** | **Parameter** | **b** | **SE** | **p** | **d** | **g** |
| **SUDS** | Intercept | 4.63 | 0.51 |  |  |  |
|  | Condition | -1.11 | 1.01 | 0.29 |  |  |
|  | Time | 0.002 | 0.50 | 1.00 |  | -0.65 |
|  | Condition x Time | -1.59 | 1.00 | 0.13 | -0.99 |  |
| **SPS** | Intercept | 1.14 | 0.12 |  |  |  |
|  | Condition | -1.00 | 0.25 | <0.001* |  |  |
|  | Time | -0.01 | 0.08 | 0.85 |  | -0.34 |
|  | Condition x Time | -0.28 | 0.16 | 0.09 | -0.41 |  |
| **FNE-B** | Intercept | 4.43 | 0.42 |  |  |  |
|  | Condition | -0.54 | 0.31 | 0.09 |  |  |
|  | Time | -0.02 | 0.11 | 0.81 |  | -0.28 |
|  | Condition x Time | -0.29 | 0.21 | 0.19 | -0.09 |  |
| **UTBAS** | Intercept | 2.72 | 0.16 |  |  |  |
|  | Condition | -0.73 | 0.32 | 0.03* |  |  |
|  | Time | -0.07 | 0.14 | 0.61 |  | -0.20 |
|  | Condition x Time | 0.15 | 0.27 | 0.59 | 0.25 |  |
| **WASSP** | Intercept | 4.00 | 0.22 |  |  |  |
|  | Condition | -0.70 | 0.43 | 0.12 |  |  |
|  | Time | -0.26 | 0.14 | 0.08 |  | -0.10 |
|  | Condition x Time | -0.38 | 0.28 | 0.18 | -0.12 |  |

Note: SUDS = Subjective Units of Distress Scale; SPS = Social Phobia Scale; FNE-B = brief version of the Fear of Negative Evaluation scale; UTBAS = Unhelpful Thoughts and Beliefs About Stuttering scale; WASSP = Wright and Ayre Stuttering Self-Rating Profile.

Supplementary table 2 – Multilevel model random effects, associated p-values and ICC

|  |  | **Pre- vs Post-treatment** | | | |
| --- | --- | --- | --- | --- | --- |
| **Measure** | **Random effect** | **Var** | **SD** | **p** | **ICC** |
| **SUDS** | Intercept | 4.31 | 2.08 | 0.002* | 0.68 |
|  | Residual | 2.00 | 1.41 |  |  |
| **SPS** | Intercept | 0.35 | 0.59 | <0.001* | 0.89 |
|  | Residual | 0.06 | 0.24 |  |  |
| **FNE-B** | Intercept | 0.39 | 0.63 | <0.001* | 0.78 |
|  | Residual | 0.10 | 0.31 |  |  |
| **UTBAS** | Intercept | 0.52 | 0.72 | <0.001* | 0.78 |
|  | Residual | 0.17 | 0.42 |  |  |
| **WASSP** | Intercept | 1.05 | 1.03 | <0.001* | 0.82 |
|  | Residual | 0.18 | 0.42 |  |  |

Note: SUDS = Subjective Units of Distress Scale; SPS = Social Phobia Scale; FNE-B = brief version of the Fear of Negative Evaluation scale; UTBAS = Unhelpful Thoughts and Beliefs About Stuttering scale; WASSP = Wright and Ayre Stuttering Self-Rating Profile.

Supplementary Table 3 – Multilevel model fit

|  | **Pre- vs. Post-treatment** | | |
| --- | --- | --- | --- |
| **Measure** | **R^2^ (fixed)** | **R^2^ (random)** | **R^2^ (total)** |
| SUDS | 0.07 | 0.64 | 0.70 |
| SPS | 0.37 | 0.53 | 0.91 |
| FNE-B | 0.38 | 0.50 | 0.88 |
| UTBAS | 0.17 | 0.62 | 0.79 |
| WASSP | 0.10 | 0.77 | 0.87 |

Note: SUDS = Subjective Units of Distress Scale; SPS = Social Phobia Scale; FNE-B = brief version of the Fear of Negative Evaluation scale; UTBAS = Unhelpful Thoughts and Beliefs About Stuttering scale; WASSP = Wright and Ayre Stuttering Self-Rating Profile.
